# Low handgrip strength is associated with worse functional outcomes in long COVID

**DOI:** 10.1038/s41598-024-52401-z

**Published:** 2024-01-24

**Authors:** Camila Miriam Suemi Sato Barros do Amaral, Cássia da Luz Goulart, Bernardo Maia da Silva, Jefferson Valente, Anna Gabriela Rezende, Eduardo Fernandes, Nadia Cubas-Vega, Mayla Gabriela Silva Borba, Vanderson Sampaio, Wuelton Monteiro, Gisely Cardoso de Melo, Marcus Lacerda, Guilherme Peixoto Tinoco Arêas, Fernando Almeida-Val

**Affiliations:** 1 Fundação de Medicina Tropical Dr Heitor Vieira Dourado, Av. Pedro Teixeira, –25 - Bairro Dom Pedro, Manaus, AM Brazil; 2 Universidade do Estado Do Amazonas, Manaus, AM Brazil; 3 Universidad Nacional Autónoma de Honduras, Tegucigalpa, FM Honduras; 4 Universidade Federal do Amazonas, Manaus, AM Brazil; 5 Hospital e Pronto-Socorro Delphina Rinaldi Abdel Aziz, Manaus, AM Brazil; 6 Instituto Todos Pela Saúde, São Paulo, SP Brazil; 7 Instituto Leônidas & Maria Deane/Fundação Oswaldo Cruz (ILMD/Fiocruz Amazônia), Manaus, Brazil

**Keywords:** Health care, Diagnosis, Diseases, Viral infection

## Abstract

The diagnosis of long COVID is troublesome, even when functional limitations are present. Dynapenia is the loss of muscle strength and power production that is not caused by neurologic or muscular diseases, being mostly associated with changes in neurologic function and/or the intrinsic force-generating properties of skeletal muscle, which altogether, may partially explain the limitations seen in long COVID. This study aimed to identify the distribution and possible associations of dynapenia with functional assessments in patients with long COVID. A total of 113 patients with COVID-19 were evaluated by functional assessment 120 days post-acute severe disease. Body composition, respiratory muscle strength, spirometry, six-minute walk test (6MWT, meters), and hand-grip strength (HGS, Kilogram-force) were assessed. Dynapenia was defined as HGS < 30 Kgf (men), and < 20 Kgf (women). Twenty-five (22%) participants were dynapenic, presenting lower muscle mass (p < 0.001), worse forced expiratory volume in the first second (FEV_1_) (p = 0.0001), lower forced vital capacity (p < 0.001), and inspiratory (p = 0.007) and expiratory (p = 0.002) peek pressures, as well as worse 6MWT performance (p < 0.001). Dynapenia, independently of age, was associated with worse FEV_1_, maximal expiratory pressure (MEP), and 6MWT, (p < 0.001) outcomes. Patients with dynapenia had higher intensive care unit (ICU) admission rates (p = 0.01) and need for invasive mechanical ventilation (p = 0.007) during hospitalization. The HGS is a simple, reliable, and low-cost measurement that can be performed in outpatient clinics in low- and middle-income countries. Thus, HGS may be used as a proxy indicator of functional impairment in this population.

## Introduction

Most people recover completely without further complications from SARS-CoV-2 infection^1^. Nonetheless, a significant subset of individuals develop unexpected symptoms weeks after infection that may persist for years^2,3^. The most reported are post-exertional malaise, fatigue, brain fog, neurocognitive impairment, dizziness, and gastrointestinal symptoms^4^. This myriad of symptoms has been collectively termed long COVID. It has become a public health problem once it is debilitating and restricts individuals from returning to daily activities, including work^5,6^. Although there is no consensus on its definition and there is no tool or biomarker that measures the condition, it is becoming increasingly well known. A global prevalence of 43% of long COVID is estimated, with a prevalence of 54% in hospitalized individuals and 34% in non-hospitalized^7^. Middle-aged, female, Hispanic and/or Latino, and economically constrained populations are at increased risk of developing long COVID^7^. The extent to which it is underdiagnosed in low-and-middle-income countries (LMICs) is poorly understood^8^.

Handgrip strength (HGS) is a well-documented proxy for muscle strength and power production. The loss of both is termed dynapenia. It is mostly associated with changes in neurologic function and/or the intrinsic force-generating properties of skeletal muscle and not with neurologic or muscular diseases^9^. Dynapenia is associated with all-cause and disease-specific mortality, as well as functional health, bone mineral density, cognitive disabilities, and depression^10^. It has also been linked to premature death in older and middle-aged individuals^11^ and young people^12^. In COVID-19 acute disease, decreased HGS is a risk factor independent of disease severity^13^ and correlates with longer hospitalization^14^.

The use of a simple and low-cost tool for the prediction of functional impairment only verified by more complex assessments, i.e., spirometry, cardiopulmonary test, and plethysmography, could be of great importance, especially in LMICs, as these tools are unavailable for most of the population. HGS, therefore, may indicate the need for further clinical investigation, specialized treatment, and rehabilitation in this population. We hypothesized that patients who experience a persistent decline in peripheral muscle strength evidenced by low handgrip strength after hospital discharge have greater alterations in respiratory function and functional capacity. Thus, this study aims to verify whether HGS is associated with functional impairment at 120 days posthospital discharge in unvaccinated individuals who had developed severe COVID-19 by early 2020. The association between HGS, respiratory function, and walked distance in the 6-min walking test (6MWT) was therefore investigated.

## Methods

### Study design and population

This was a longitudinal study carried out between April and October 2020 at the *Hospital e Pronto-Socorro Delphina Rinaldi Abdel Aziz*, in Manaus, Western Brazilian Amazon. Hospitalized. COVID-19 patients were followed up after hospitalization and performed pulmonary function and physical capacity tests 120 days after discharge (D120). Individuals aged 18 years or older, of both sexes and positive for SARS-CoV-2 by RT-qPCR at hospitalization were included.

### Procedures performed at the follow-up visit (D120)

The D120 visit consisted of assessing body composition, HGS, and respiratory function, with a pulmonary function test (spirometry), respiratory muscle strength (RMS), and functional capacity using the 6MWT. Test details are presented below. All data were recorded online in an electronic medical recording system (Medview version 710801 and Esthor) and then registered in an electronic database (REDCap). All procedures were performed by harmonized and protocol-trained study staff.

#### Handgrip strength and dynapenia case definition

Three measurements were performed using a digital hand-held dynamometer (Instrutherm®, Brazil), standing position for each hand, at 1-min intervals, with the elbow straight and fully extended, alternating between dominant and non-dominant sides. The maximum value was derived for each arm, while HGS was defined as the highest value of the six attempts. Dynapenia was considered when participants presented cut-off values of < 30 Kgf (kilogram-force) for men and < 20 Kgf for women^15^. Handgrip measurement has the advantage of being simple and easy and of being used as an index of whole-body muscular strength^16^.

#### Pulmonary function and respiratory muscle strength (RMS)

Spirometry (microQuark, Cosmed®, Italy) was performed to determine pulmonary function. Briefly, forced vital capacity (FVC, liters), forced expiratory capacity at the first second of exhalation (FEV_1_, liters) and FEV_1/_FVC were assessed. The predicted values were calculated according to the ERS equation^17^. The RMS was evaluated using a digital manometer (MDI, MVD300, Brazil). Both tests were performed according to the *American Thoracic Society* and *European Respiratory Society* guidelines^18^. Maximum inspiratory pressure (MIP, cmH_2_0) was obtained after the individual expired to residual volume and performed a maximal effort inspiration against a closed valve, during which the pressure was measured. For the assessment of maximum expiratory pressure (MEP, cmH_2_0), patients underwent an inspiration up to full lung capacity, followed by a maximal effort expiration against a closed valve for two seconds, after which the valve was opened. The reference values were defined by the Neder equation^19^. Those who presented altered blood pressure levels and those with poor-quality spirometry were not included in the analysis.

#### Functional capacity and body composition

For the 6MWT, subjects were instructed and encouraged (standardized verbal encouragement) to walk the longest (meters) possible distance in six minutes on a 30-m-long flat corridor. The test was performed according to the *American Thoracic Society* recommendations^20^. We used the percentage of the predicted value for the Brazilian population^21^. The assessment of body composition was performed using a previously calibrated digital full-body bioimpedance scale (HBF-514 OMRON®, Japan) and was carried out with the participants standing barefoot and upright. Individuals were on a fasting regime at the time of assessment. Weight (Kg), body mass index (BMI), muscle mass (Kg), and fat mass (Kg) variables were extracted. The height of the participants was collected using a standard measuring tape divided into centimeters, keeping them barefoot and with their bodies upright.

### Statistical analysis

Participants were grouped according to the HGS test and further stratified into dynapenic and non-dynapenic to verify whether HGS was associated with functional impairment in long COVID. The Shapiro–Wilk test was used to verify the data distribution. Descriptive data are shown as mean and standard deviation (SD), or median and interquartile range (IQR), when applicable. The Mann–Whitney test was used to compare both groups (i.e., Dynapenia *vs.* non-dynapenia). For categorical variables, the chi-square test was applied. Correlation analysis was performed with Spearman´s test. A regression model was used to determine the influence of age associated with dynapenia on functional and clinical variables. All tests were performed using R software version 4.3.0 and p-values ≤ 0.05 were considered statistically significant.

### Ethical aspects

The Brazilian Committee on Ethics in Human Research approved the study (CAAE 30615920.2.0000.0005). This study was conducted following the Declaration of Helsinki principles and the Good Clinical Practice guidelines of the International Conference on Harmonization. All hospitalized patients were informed about the study objectives and risks, after which they were invited to participate. All individuals were given time to carefully read and sign an informed consent form.

## Results

A total of 113 participants were included in this study, with a median age of 48 years old, and 54% were female. A total of 22% had dynapenia (Table 1).

**Table 1:** Characterization at D120 of the total sample and non-Dynapenia vs Dynapenia in long-Covid patients.

Variables	Total (n = 113)	Non-Dynapenia (n = 88)	Dynapenia (n = 25)	P value
Age, years, median (IQR)	48 (41–58)	47 (40–54)	57 (51–63)	0.002
Gender, woman, n (%)	61 (54)	50 (56.8)	11 (44.2)	0.26
Allocation at hospitalization, IMP, n (%)	55 (48.7)	42 (47.7)	13 (52)	0.71
BMI (kg/m^2^), medium (IQR)	33.2 (31.9–34.4)	33.7 (32.3–35.0)	31.4 (28.5–34.3)	0.09
Muscle Mass (kg), medium (IQR)	23.9 (18.2–29.3)	26.1 (20.0–29.7)	20.6 (16.0–23.2)	< 0.001
Fat Mass, (Kg) medium (IQR)	31.9 (25.4–41.7)	32.7 (26.7–42.5)	28.9 (20.5–38.7)	0.202
HGS (Kg), medium (IQR)	30.2 (24.0–39.8)	34.0 (26.6–43.7)	18.5 (15.0–26.8)	< 0.001
Cough, n/N (%)	99/112 (88.4%)	79/88 (89.8%)	20/24 (83.3%)	0.38
Myalgia, n/N (%)				0.07
No	27/112 (24.1%)	18/88 (20.5%)	9/24 (37.5%)	
Yes	84/112 (75.0%)	69/88 (78.4%)	15/24 (62.5%)	
Unknown	1/112 (0.9%)	1/88 (1.1%)	0/24 (0.0%)	
Fatigue, n (%)	101/112 (90.2%)	81/88 (92.0%)	20/24 (83.3%)	0.20
Nausea, n (%)	62/112 (55.4%)	51/88 (58.0%)	11/24 (45.8%)	0.29
Vomiting, n (%)	44/112 (39.3%)	38/88 (43.2%)	6/24 (25.0%)	0.11
Diarrhea, n (%)	71/112 (63.4%)	56/88 (63.6%)	15/24 (62.5%)	0.92
Anosmia, n (%)	91/112 (81.3%)	75/88 (85.2%)	16/24 (66.7%)	0.03

*IMP* intravenous methylprednisolone, *BMI* body mass index, *IQR* interquartile range, *HGS* handgrip strength, *Kg* kilograms; Applied Mann–Whitney test.

The baseline demographic, clinical, laboratory, and radiological findings of the study population on the first day of hospitalization during acute severe COVID-19 are presented in Table 2. Those with dynapenia presented significantly lower muscle mass compared to the non-dynapenic group, 20.6 kg and 26.1 kg (p < 0,001), respectively, at the D120 follow-up visit. As expected, the HGS was significantly lower in the dynapenia group compared to the non-dynapenic group, 18.5 Kgf and 34.0 Kgf (p < 0.001), respectively, at D120. Patients with dynapenia had higher ICU admission rates (19% *vs* 2.9%, p = 0.010) and need for invasive mechanical ventilation (16% *vs* 2.3%, p = 0.007) than patients without dynapenia. The BMI (p = 0.04), the proportion of individuals with a smoking history (p < 0.01), and diabetes (p = 0.01) were higher in the dynapenia group (Table 2).

**Table 2:** Baseline demographic, clinical, laboratory, and radiological findings of the study population on the first day of hospitalization during acute COVID-19.

	Total (n = 113)	Non-Dynapenia (n = 88)	Dynapenia (n = 25)	p-value
BMI, kg/m^2^, median (IQR)	30.7 (27.1–35.5)	31.4 (27.9–35.7)	28.7 (23.8–34.2)	0.04
Smoking history, n (%)	30/108 (27.8%)	16/83 (19.3%)	14/25 (56.0%)	< 0.01
Alcohol use disorder, n (%)	31/113 (27.4%)	25/88 (28.4%)	6/25 (24.0%)	0.66
Diabetes, n (%)	27/113 (23.9%)	16/88 (18.2%)	11/25 (44.0%)	0.01
COPD, n (%)	8/108 (7.4%)	6/83 (7.2%)	2/25 (8.0%)	0.90
Hypertension, n (%)	39/113 (34.5%)	28/88 (31.8%)	11/25 (44.0%)	0.26
ICU on admission, n (%)	6/89 (6.7%)	2/68 (2.9%)	4/21 (19.0%)	0.01
IMV on admission, n (%)	6/113 (5.3%)	2/88 (2.3%)	4/25 (16.0%)	0.01
Oxygen saturation (SpO_2_), n (%)	97.0 (95.0–98.0)	96.5 (95.0–98.0)	97.0 (95.0–98.0)	0.91
Creatine kinase, U/L, median (IQR)	76.3 (39.9–144.1)	84.5 (40.1–142.3)	73.4 (38.4–174.5)	0.76
Creatine kinase MB, U/L, median (IQR)	18.3 (12.0–23.1)	17.7 (12.0–21.7)	20.4 (17.5–23.6)	0.37
LDH, U/L, median (IQR)	572.0 (293.0–892.0)	494.5 (302.5–858.5)	773.0 (254.0–992.0)	0.77
D-dimer, ng/mL, median (IQR)	641.3 (458.0–1576.2)	593.4 (456.9–1141.5)	1576.2 (571.6–4066.0)	0.13
C-reactive protein, mg/L, median (IQR)	66.7 (33.6–87.3)	66.6 (27.9–92.2)	67.7 (45.9–75.2)	0.63
IL-6, pg/mL, median (IQR)	33.7 (8.3–82.2)	27.6 (6.3–81.0)	46.8 (23.3–105.9)	0.06
Ground-glass opacity infiltration, n (%)	80/85 (94.1%)	60/64 (93.8%)	20/21 (95.2%)	0.80
Consolidation, n (%)	75/85 (88.2%)	56/64 (87.5%)	19/21 (90.5%)	0.71
Unilateral consolidation, n (%)	7/85 (8.2%)	5/64 (7.8%)	2/21 (9.5%)	0.80
Bilateral consolidation, n (%)	68/85 (80.0%)	51/64 (79.7%)	17/21 (81.0%)	0.90
Hematocrit, %, median (IQR)	40.6 (38.3–43.1)	40.7 (38.6–43.4)	39.6 (35.2–42.5)	0.10
Hemoglobin, g/dL, median (IQR)	12.6 (11.8–13.4)	12.8 (11.9–13.5)	12.6 (11.0–13.3)	0.20
qSOFA score > = 2, N (%)	16/113 (14.2%)	10/88 (11.4%)	6/25 (24.0%)	0.11
Pleural effusion, n (%)	18/85 (21.2%)	12/64 (18.8%)	6/21 (28.6%)	0.34

*COPD* chronic pulmonary obstructive disease, *LDH* lactate dehydrogenase, *SD* standard deviation, *ICU* intensive care unit, *IMV* invasive mechanical ventilation, *BMI* body mass index, *BFM* body fat mass, *BMM* body muscle mass, *kg* kilogram, *m* meter, *IQR* interquartile range, *IL* interleukin.

When comparing muscle mass (kg) on D1 versus D120 in dynapenic versus non-dynapenic groups, we found a significant reduction after 120 days in the dynapenic group [D1 dynapenia: 30.7 (37–23) *versus* D120 dynapenia: 19.9 (22–17) p < 0.001].

Dynapenic individuals presented worse forced expiratory volume in the first second (FEV_1_, liters) (p < 0.001) and forced vital capacity (FVC, liters) (p < 0.001), reduced MIP (p = 0.007) and MEP (p = 0.002) pressures, significantly reduced walking distance (p < 0.001) and percentage of predicted walking distance (p = 0.001) on the 6MWT compared to non-dynapenic patients (Table 3).

**Table 3:** Spirometry, RMS, and functional capacity in long COVID patients (day 120).

Variables	Total (n = 113)	Non-Dynapenia (n = 88)	Dynapenia (n = 25)	*p*-value
Spirometry				
FVC (L)	3.1 (2.4–3.5)	3.3 (2.5–3.7)	2.3 (2.0–3.0)	< 0.001
FVC % of predicted	89 (85–92)	88 (83–92)	90 (81–99)	0.911
FEV_1_ (L)	2.5 (1.9–3.0)	2.7 (2.0–3.1)	2.0 (1.6–2.4)	< 0.001
FEV_1_% of predicted	87 (83–90)	85 (81–90)	88 (80–97)	0.691
FEV_1_/FVC (%)	82.7 (77.5–85.3)	83.4 (77.1–85.5)	80.5 (77.5–83.4)	0.24
Respiratory muscle strength				
MIP (cmH_2_O)	91.0 (73.0–113.0)	92.5 (79.0–115.5)	73.0 (51.0–105.0)	0.007
MIP (%)	70.9 (62.1–76.2)	72.6 (68.3–77.0)	64.8 (51.2–78.3)	0.148
MEP (cmH_2_O)	108.0 (82.0–132.0)	111.0 (88.0–138.0)	87.0 (67.0–108.0)	0.002
MEP(%)	102.4 (91.1–101.4)	104.3 (98.4–110.3)	96.8 (85.2–100.1)	0.235
Functional capacity				
6MWD (m)	395.0 (330.0–456.0)	417.0 (353.0–468.0)	336.0 (275.0–370.0)	< 0.001
6MWD (%)	542.9 (471.7–596.2)	557.5 (503.3–604.5)	471.7 (432.6–531.9)	< 0.001

*FVC* forced capacity vital, *FEV*_*1*_ forced expiratory volume in the first second; MIP: Maximum inspiratory pressure; MEP: Maximum expiratory pressure; 6MWD: six-minute walking test. Applied Mann–Whitney test.

**Figure 1:**
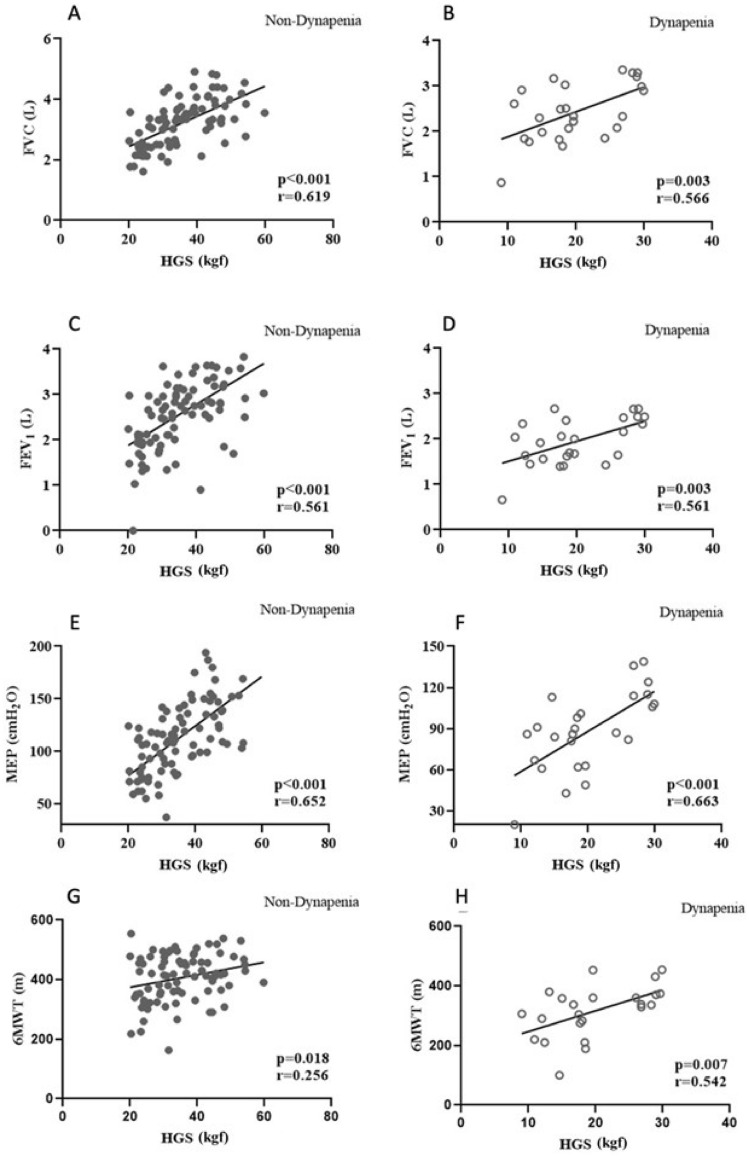
Spearman correlation between HGS and functional outcomes: The correlation analysis revealed significance between HGS and the walked distance for all participants, which also remained significant among both groups separately.

The correlation analysis revealed significance between HGS and walked distance for all participants (*rho* = 0.450, p < 0.001), which also remained significant among both groups separately (Fig. 1). Regression analyses revealed a significant association between HGS and FEV_1_ (p < 0.001, CI 95% − 0.0728 − 0.0377), MEP (p < 0.001 CI 95% − 0.0016 − 0.008), and 6MWT (p < 0.001 CI 95% − 0.0017 − 0.0015), even after controlling for age, R2 = 0.22 (Table 4).

**Table 4:** Regression analyses between HGS and functional outcomes, with age.

Dynapenia	Coef	P > z	95% CI	
FEV_1_ (%)	− 0.05	0.00	− 0.07	− 0.03
MIP (cmH_2_O)	0.00	0.62	− 0.00	0.00
MEP (cmH_2_O)	− 0.00	0.00	− 0.00	− 0.00
6MWT (D)	− 0.00	0.00	− 0.00	− 0.00

*FEV*_*1*_ forced expiratory volume in the first second; MIP: Maximum inspiratory pressure; MEP: Maximum expiratory pressure; 6MWT: six minutes walking test distance. R2 = 0.22.

## Discussion

This study showed that an important proportion of individuals with long COVID have decreased HGS. In addition, dynapenia was associated with worse lung function test scores and lower respiratory muscle strength, which ultimately negatively impacted the walked distance on the 6MWT after 120 days from hospitalization. Despite a significant and expected higher age in the dynapenic group, the HGS was associated with lower FEV_1_, MEP, and 6MWT, even when controlling for age. HGS was correlated significantly with the walked distance on the 6MWT and with worse pulmonary function in these individuals.

After 120 days, 22% of the participants were dynapenic and still had a loss of muscle strength, which was reflected in decreased muscle functioning. The skeletal muscle’s ability to produce strength (i.e., maximal voluntary force) and power (strength *vs* speed) affects health outcomes and is associated with increased disability, becoming an important component of health and disease for all ages. Being referred to as an age-related loss of skeletal muscle strength and power, dynapenia is less associated with muscle mass status, with evidence pointing to a mitochondrial dysfunction-associated reduction in ATP generation capacity, which ultimately compromises tissue homeostasis^22^. The consequences, therefore, of reduced grip strength are increased risk for physical disability^23,24^, poor physical performance and death from cardiovascular and non-cardiovascular causes, and stroke^25–27^.

The current literature shows that most of the symptoms of long COVID occur in individuals who have been hospitalized, but also in non-hospitalized individuals, or severe and non-severe COVID-19^28^, with more severe individuals having worse outcomes six months after hospital discharge. Given the number of individuals who recovered from acute COVID-19, regardless of the acute disease phenotype, long COVID is a problem that is far from being fully understood. Most sequelae seem to resolve within six months^29^, but in some individuals, it may persist for years^30^. The individuals from this study demonstrated a worrying functional outcome after four months of acute disease, which probably limited their daily activities. Further studies are needed to see if these alterations persist for longer periods and how they reverberate in day-to-day and labor activities.

A significant proportion of individuals with diabetes, previous smoking habits, lower BMI, older age, and in need for ICU and IMV at hospital admission presented reduced grip strength 120 days after hospital discharge. It is important to emphasize that a 1% loss of lean mass is equivalent to a 3% reduction in muscle strength in the elderly, which may have an impact on physical performance and function^31^. To what extent does the interplay between hospitalization (disuse, hypoxemia, malnutrition, sedation, pharmacological therapy, etc.), past medical history and lifestyle habits, and inflammation and immune activation from SARS-CoV-2 acute infection induce muscle wasting, further reducing muscle function acutely and chronically are not completely understood and merit future investigation.

Studies on respiratory muscles have also shown negative outcomes. Formenti et al.^32^ evidenced significantly reduced echogenicity in right and left intercostal and diaphragm muscles at 24 h at ICU in deceased COVID-19 patients in comparison to those who survived, indicating loss of muscle mass^32^. Regmi and cols. reported persistence of diaphragm muscle weakness 15 months after hospitalization for COVID-19^33^. In this study spirometry parameters, RMS, and 6MWT were all reduced among those with reduced handgrip strength several months after hospital discharge, corroborating these previous findings.

Furthermore, chronic fatigue, post-exertional malaise, exercise intolerance, and other functional abnormalities increasingly observed in long COVID remain poorly understood. A study on the histopathological changes in 16 long COVID individuals presenting fatigue more than a year after the acute infection showed muscle fiber atrophy and regeneration in 38% and 56%, respectively^34^. Also, 62% of these individuals presented loss of cytochrome c oxidase activity and other alterations compatible with mitochondrial changes, together with T lymphocytes and/or muscle fiber human leukocyte antigen ABC expression and capillary basal lamina injury, which, according to the authors, may be involved in reduced energy supply and fatigue^34^.

Research on causes for long COVID has narrowed some hypotheses for such conditions, which include the persistence of SARS-CoV-2 reservoirs, presence of microthrombi, induction and persistence of autoantibodies, hyperreactive immune activation, reactivation of other latent viral coinfections such as Epstein-Barr Virus (EBV), mitochondrial dysfunction, and gut dysfunction/dysbiosis, that may act individually or collectively, producing this syndrome^35^.

Recently, it has been reported that acute inflammation, associated with COVID-19, is a potent detrimental stimulus for the development of reduced muscle strength and mass^36,37^. Of the various deleterious influences of inflammation, elevated c-reactive protein, and interleukin-6 have been strongly correlated with reduced muscle strength and frailty^36,37^. Also, mitochondrial dysfunction and oxidative stress have been associated with the development of dynapenia^22,38^. Both these physiological disturbances have also been associated with long COVID pathogenesis^39^. We found that patients with long COVID with dynapenia had reduced muscle mass, which was reflected in the handgrip strength, exercise capacity by the 6MWT, and respiratory muscle strength. Therefore, reduced muscle mass and the ability to produce strength, as seen in the dynapenic group, reflected on muscle function systemically, as seen in the reduced RMS and spirometry tests, which may reflect a multifactorial origin to decreased muscle functional features globally, highlighting the possible multifactorial aspect of chronic disability in this population. One of the hypotheses is that these patients present dynapenia at hospitalization and chronically due to an imbalance between muscle protein synthesis and muscle protein breakdown maintained during the follow-up period of 120 days.

The HGS was associated with respiratory function, respiratory muscle pressure, and physical capacity measured in the 6MWT in this study. Several studies have demonstrated the association between the severity of acute COVID-19 and functional outcomes, such as 6MWT, lung diffusion capacity, and spirometry months later^40,41^, which are interesting tools for assessing the detrimental consequences of this disease^42^. Nonetheless, the association between HGS and these parameters has important practical implications in long COVID diagnosis and care once a great number of individuals do not have access to complex functional assessments and remain without proper functional diagnosis and quantitative assessments for exercise prescription on an individual basis and, therefore, being withheld of efficient rehabilitation. Additionally, it would be of great importance to establish cut-off points for such tests, enabling healthcare practitioners to better assess and manage this population, as occurs in other diseases, since HGS is quick, safe, and cheaper^16^.

Early evidence of corticosteroid therapy for COVID-19 showed positive effects on mortality outcomes^43^, but optimal dosing is still under research^44^. However, indistinct use of corticosteroids, acute or chronically, may lead to several adverse events, one of them being myopathy, which could be difficult to distinguish from COVID‐19‐related critical illness neuropathy and myopathy^45^, or post-ICU syndrome and ICU-acquired weakness, which are well-recognized conditions^46^. Corticosteroid therapy is a common cause of non-inflammatory myopathy, ultimately causing muscle weakness, which may last from weeks to months if use is persistent^47^. Also, in humans, corticosteroids are known to affect both respiratory and limb muscles, causing loss of muscle cross-sectional area, inducing weakness, and wasting, and ultimately leading to loss of function^47^. Nonetheless, these factors altogether may likely explain to some extent acute weakness and fatigue in severe and hospitalized COVID-19 individuals. Their association with persistent long-term outcomes needs further research.

The present study demonstrates the association of a simple tool, such as HGS, with other functional outcomes that demand more trained personnel and robust equipment, such as spirometry and 6MWT, in individuals who had severe COVID-19 in the early 2020s, when the predominant SARS-CoV-2 lineages were B.1.195, B1.1.33 and B.1.1.28, before P.1, Delta and Omicron^48^. Also, these individuals were not vaccinated at the time, which likely worsened acute disease and long-term outcomes, as increasing evidence shows the association between prior vaccination and the reduction of long COVID^49^. Nonetheless, the individual characteristics of the population of this study represent an important proportion of the population lingering with symptoms from the COVID-19 infection back then and still suffering from long COVID.

This study has some limitations. The sample is relatively small and there is a short-term follow-up. There was no assessment of HGS and other functional outcomes at hospitalization, or at hospital discharge, which impairs longitudinal comparisons between these variables. This is a single-center study with individuals infected by SARS-CoV-2 in the early 2020s, so the results presented here may not be directly extrapolated to the long-term health functional outcomes of those individuals infected with more recent variants of the virus.

## Conclusions

Low handgrip strength represents poor health and carries a poor prognosis. We demonstrated in patients with long COVID that dynapenia is associated with changes in lung function, respiratory muscle strength, and exercise capacity and that in-hospital outcomes might be directly related to muscle strength months later. HGS is a simple, reliable, and low-cost measurement that can be performed as a proxy for functional impairment in outpatient clinics and primary care facilities. Acknowledging the importance of recognizing this condition could promptly help in patient stratification and risk prevention of patients with long COVID, which may reduce the development of comorbidities, delay the functional decline, improve the prognosis and survival of these patients, and accelerate the return to daily and work-related activities. This is of interest for low and middle-income countries, which suffer from economic constraints in general, with a great part of the population without proper access to healthcare. Demonstrating the importance of identifying such functional impairments easily enables healthcare providers to properly refer patients to rehabilitation services with a focus on improving muscle strength, pulmonary function, and functional capacity. This could be one simple, yet robust, primary healthcare practice tool for timely screening and better managing long COVID patients.

## Data Availability

The original contributions presented in the study are included in the article. The raw data supporting the conclusions of this article shall be made available upon reasonable request to the corresponding author.
